# Elements of Immunoglobulin E Network Associate with Aortic Valve Area in Patients with Acquired Aortic Stenosis

**DOI:** 10.3390/biomedicines9010023

**Published:** 2020-12-31

**Authors:** Daniel P. Potaczek, Aleksandra Przytulska-Szczerbik, Stanisława Bazan-Socha, Artur Jurczyszyn, Ko Okumura, Chiharu Nishiyama, Anetta Undas, Ewa Wypasek

**Affiliations:** 1 Translational Inflammation Research Division & Core Facility for Single Cell Multiomics, Medical Faculty, Philipps University Marburg, Member of the German Center for Lung Research (DZL) and the Universities of Giessen and Marburg Lung Center, 35043 Marburg, Germany; danppot@gmail.com; 2 Krakow Center for Medical Research and Technology, John Paul II Hospital, 31-202 Krakow, Poland; a.przytulska@szpitaljp2.krakow.pl (A.P.-S.); mmundas@cyf-kr.edu.pl (A.U.); 3 Department of Internal Medicine, Jagiellonian University Medical College, 31-066 Krakow, Poland; mmsocha@cyf-kr.edu.pl; 4 Department of Hematology, Jagiellonian University Medical College, 31-501 Krakow, Poland; mmjurczy@cyf-kr.edu.pl; 5 Atopy Research Center, Juntendo University School of Medicine, Tokyo 113-8421, Japan; kokumura@juntendo.ac.jp; 6 Laboratory of Molecular Biology and Immunology, Department of Biological Science and Technology, Tokyo University of Science, Tokyo 125-8585, Japan; chinishi@rs.tus.ac.jp; 7 Institute of Cardiology, Jagiellonian University Medical College, 31-202 Krakow, Poland; 8 Faculty of Medicine and Health Sciences, Andrzej Frycz Modrzewski Krakow University, 30-705 Krakow, Poland

**Keywords:** aortic valve area (AVA), aortic (valve) stenosis (AS; AVS), FcԑRI α-subunit (FcԑRIα) gene (*FCER1A*), (genetic) polymorphism, high-affinity IgE receptor (FcԑRI), immunoglobulin E (IgE)

## Abstract

Allergic mechanisms are likely involved in atherosclerosis and its clinical presentations, such as coronary artery disease (CAD). It has been previously reported that CAD severity associates with serum levels of immunoglobulin E (IgE), the molecule that, along with its high-affinity receptor (FcԑRI), plays a central role in allergic reactions. Considering multiple pathophysiological similarities between atherosclerosis and acquired aortic (valve) stenosis (AS), we speculated that allergic pathways could also contribute to the AS mechanisms and grading. To validate this hypothesis, we first checked whether total serum IgE levels associate with echocardiographic markers of AS severity. Having found a positive correlation between serum IgE and aortic valve area (AVA), we further speculated that also total IgE-determining genetic polymorphisms in *FCER1A*, a locus encoding an allergen-biding FcԑRI subunit, are related to acquired AS severity. Indeed, the major allele of rs2251746 polymorphism, known to associate with higher IgE levels, turned out to correlate with larger AVA, a marker of less severe AS. Our findings surprisingly suggest a protective role of IgE pathways against AS progression. IgE-mediated protective mechanisms in AS require further investigations.

## 1. Introduction

The immune system plays an essential role in the development and progression of atherosclerosis and related diseases, including coronary artery disease (CAD), peripheral artery disease, and stroke. Moreover, there is a growing body of evidence for the contribution of allergic mechanisms, such as those related to the immunoglobulin E (IgE) network, to the pathogenesis of atherosclerosis [1,2,3]. The IgE network comprises proteins associated with IgE activity, including its two principal receptors, the high- (FcԑRI) and the low-affinity IgE receptors [4,5].

Previous studies have demonstrated that the severity of CAD associates with total serum IgE levels [6,7,8]. In turn, atherosclerosis shares multiple pathophysiological features and risk factors with aortic (valve) stenosis (AS) [9,10,11], the most common acquired heart valve disease, and another chronic vascular inflammatory disorder [9,12]. Therefore, we hypothesized that AS severity may also be related to the IgE network. To verify this speculation, we sought to investigate the associations between AS severity and total serum IgE concentrations as well as the polymorphisms in the gene encoding the α-subunit of FcԑRI (*FCER1A*) representing the two tagging bins constituting the primary total serum IgE susceptibility loci [13,14,15,16].

## 2. Experimental Section

### 2.1. Study Subjects

A total of 115 consecutive patients with acquired AS, recruited between March 2015 and November 2017, represented the original study group, in which total serum IgE levels were assessed (Table S1). The Jagiellonian University Ethical Committee approved the study, and all the participants provided their written informed consent. AS was defined as a mean transvalvular gradient greater than or equal to 40 mm Hg and/or aortic valve area (AVA) less than 1 cm^2^ on the basis of transthoracic echocardiography, performed by an experienced cardiologist using a Vivid 7 ultrasound equipment (GE Healthcare, Chalfont St Giles, UK).

For the purposes of the genetic association study of AS severity, we enriched the original group with an additional 305 acquired AS patients, recruited for our previous studies between September 2009 and May 2012 [17,18], thus forming the expanded study group of 420 subjects in total (Table S2).

All patients were originally referred to either the Department of Cardiology, John Paul II Hospital, or the Department of Cardiovascular Surgery and Transplantation, Jagiellonian University School of Medicine, both in Krakow, Poland, for further diagnostic work-up, and all came from the Malopolska region in the south of Poland. The exclusion criteria were acute infection; Valsalva sinus aneurysm or rheumatic AS; known cancer; autoimmune or allergic disorders; endocarditis; previous cardiac surgery; liver failure with an alanine aminotransferase above the upper limit of the reference range; and a history of myocardial infarction (MI), stroke, venous thromboembolism, or bleeding.

### 2.2. Laboratory Tests

Total serum IgE was assayed using ELISA (Biokom, Janki k/Warszawy, Poland). C-reactive protein (CRP) was determined by immunoturbidimetry (Roche Diagnostics, Mannheim, Germany).

### 2.3. Genotyping

DNA was extracted from whole blood or a buffy coat using the NucleoSpin Blood DNA Kit (Macherey-Nagel, Düren, Germany) according to the manufacturer’s protocol. *FCER1A* rs2252226 and rs2251746 variants were genotyped for the purposes of the present study using C_15886139_10 and C_1840470_20 TaqMan SNP Genotyping Assays (Applied Biosystems, Thermo Fisher Scientific, Foster City, CA, USA), respectively. Genotyping methodology has been validated in a control group comprising healthy individuals, as previously reported [19]. Both polymorphisms were successfully genotyped in AS patients with a call rate of about 98% and their genotypes remaining in Hardy–Weinberg equilibrium (HWE; Table S3).

### 2.4. Statistical Analysis

The distribution of the continuous variables was analyzed with Shapiro–Wilk test. Those normally distributed are presented as mean ± standard deviation. Variables with the distribution different from normal are, if not otherwise indicated, given as median [interquartile range] and were analyzed with Mann–Whitney test or Kruskal–Wallis test (and then, if required, also with Jonckheere–Terpstra trend test). Adjustments for potential categorical and continuous confounders were performed using analysis of covariance (ANCOVA), with variables having distribution different from normal subjected to square-root transformation before entering the model. Associations between two continuous variables were calculated using Spearman’s coefficient of rank correlation. Adjustments for potential categorical and continuous confounders were conducted using least squares multiple regression, with variables having distribution different from normal subjected to square-root transformation before entering the model. Conformity with HWE was tested with the chi-squared goodness-of-fit test. Statistical calculations were performed using MedCalc Version 18.10 (MedCalc Software, Ostend, Belgium) and Dell Statistica 13 (Dell, Round Rock, TX, USA) programs.

## 3. Results

### 3.1. Correlations between Total Serum IgE Levels and Echocardiographic Parameters in Acquired AS Patients

**Figure 1:**
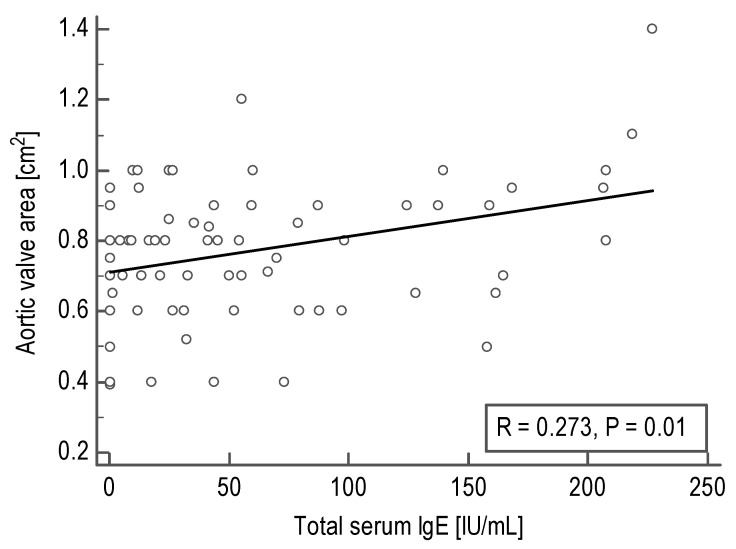
Correlation between aortic valve area and total serum immunoglobulin (IgE) levels in acquired aortic stenosis patients calculated using Spearman’s coefficient of rank correlation (*R*).

First, we sought to investigate whether the echocardiographic parameters correlate with total serum IgE concentrations in AS patients. Although a mean or maximum gradient and left ventricular ejection fraction were not associated with total serum IgE, we documented a positive correlation between IgE and aortic valve area (AVA; *R* = 0.27, *P* = 0.01; Table 1, Figure 1). This correlation remained significant also after adjustment for age, sex, BMI, and CRP levels (*P* <0.05).

### 3.2. Association between FCER1A Polymorphisms and AVA in Acquired AS Patients

**Figure 2:**
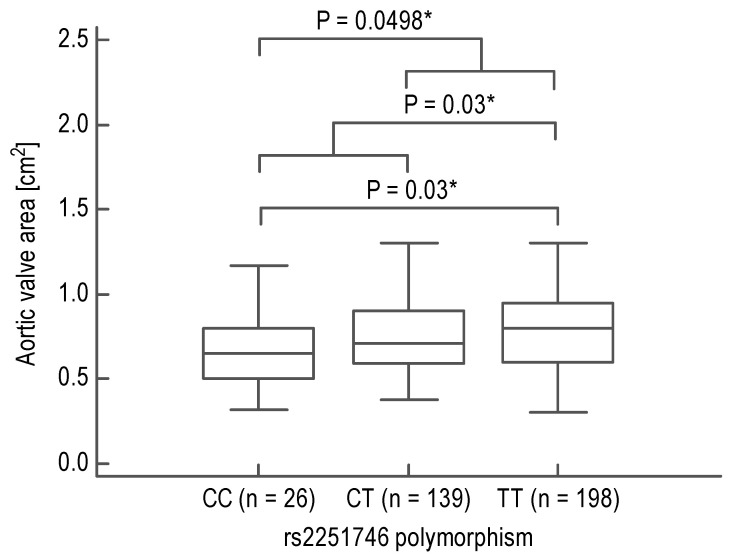
Median aortic valve area in acquired aortic stenosis patients having different genotypes of the high-affinity immunoglobulin E receptor α-subunit locus (*FCER1A*) rs2251746 polymorphism. Boxes range from the 25th to the 75th percentile with a horizontal line at the median and vertical lines extending to the 10th and 90th percentiles. *P*-values calculated using Mann–Whitney test are given. *P*-values of less than 0.05 are marked with a star (“*”).

In the second step, in a large group of 420 AS subjects, we analyzed the relationships between *FCER1A* polymorphisms and AVA. The rs2252226 polymorphism was not associated with AVA (TT, *n* = 122, 0.73 [0.56–0.93]; TC, *n* = 177, 0.72 [0.60–0.95]; and CC, *n* = 64, 0.79 [0.59–0.90]; *P* = 0.91). However, the rs2251746 polymorphism demonstrated a significant association with AVA (TT, *n* = 198, 0.80 [0.60–0.95]; TC, *n* = 139, 0.71 [0.59–0.90]; and CC, *n* = 26, 0.65 [0.50–0.80]; *P* = 0.04, *P*_trend_ = 0.02), which remained significant after adjustment for age, sex, BMI, and CRP levels (*P* = 0.02). Moreover, contrasting minor or major homozygotes with the reminder as well as opposite homozygotes with each other also yielded significant results (*P* = 0.0498, *P* = 0.03, and *P* = 0.03, respectively; Figure 2).

### 3.3. The Effects of FCER1A Polymorphisms on Total Serum IgE in Acquired AS Patients

Finally, we investigated whether *FCER1A* polymorphisms associated with total IgE in AS subjects. The rs2251746 polymorphism tended to be associated with total serum IgE (*P* = 0.09), with the highest values in TT and the lowest in CC homozygotes (Table S4). Furthermore, contrasting carriers of the T allele or TT homozygotes with CC homozygotes demonstrated lower IgE in the latter group (*P* = 0.03 in both; Table S4). In turn, although TT homozygotes of the rs2252226 polymorphism had the lowest and CC homozygotes the highest total serum IgE values, the differences were not significant (Table S4). Only a tendency towards an association was observed when TT homozygotes were contrasted with the carriers of the C allele (*P* = 0.07; Table S4).

## 4. Discussion

To our knowledge, relationships between IgE network and acquired AS severity have not been investigated thus far. However, on the basis of the mechanistic similarities between AS and CAD [9,10], as well as previously reported correlations between the severity of CAD and IgE levels [6,7,8], we speculated that similar relationships could also be observed in AS.

Indeed, we found significant and robust associations between the elements of the IgE network and AS severity, but their directions were opposite to what we had originally expected. Specifically, larger AVA indicating less advanced AS [20] correlated with increased serum IgE concentrations. Furthermore, the major allele of the rs2251746 polymorphism, known to be strongly associated with elevated total serum IgE levels [13,14,15,16], was related to the higher AVA. These are intriguing and unexpected findings requiring a comment.

**Figure 3:**
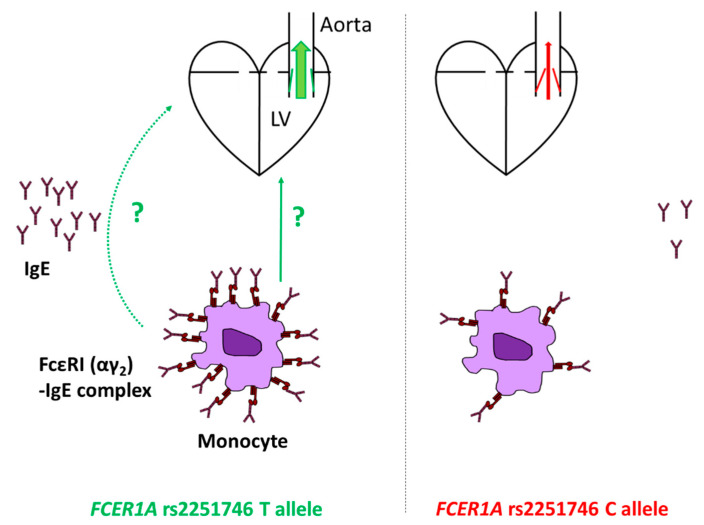
Schematic illustration of the finding and hypothetical mechanism(s) underlying it. The T allele of the high-affinity immunoglobulin E (IgE) receptor (FcԑRI) α-subunit (FcԑRIα) gene (*FCER1A*) rs2251746 polymorphism is associated with larger aortic valve area and thus less severe aortic valve stenosis. The mechanism behind it is unknown but might involve monocytes and their enhanced functions resulting from higher surface FcԑRI(α) expression. Serum IgE might also contribute or be a bystander with potential marker properties. LV denotes left ventricle.

An increase in FcεRI expression might lead to higher concentrations of IgE in serum, for instance, through the stabilizing effect of mast cell or basophil receptor on IgE upon binding [21,22]. This might mechanistically explain the association of the major allele of the rs2251746 polymorphism [4], shown to increase *FCER1A* promoter activity and thus FcεRI(α) expression [23,24], with higher total IgE in serum. Hence, our current results might suggest that higher total serum IgE and/or cell surface FcεRI expression levels protect against the development of more severe forms of AS. Mechanisms behind that effect remain unclear, particularly because numbers of mast cells in pathologically altered aortic valve tissue in AS correlate positively with disease severity, thus inversely with AVA [25]. However, it has been reported that cardiac mast cells are activated directly predominantly by noxious metabolic agents or neuropeptide substance P via MAS-related G protein-coupled receptor-X2 (MRGPRX2) and not through the IgE–FcεRI-dependent pathway [26,27,28,29,30]. Therefore, one might speculate that other FcεRI-expressing cell types might contribute to the protective role of the IgE network in AS progression, such as monocytes or macrophages. Monocytes play an essential role in each AS cardinal component, including inflammation, fibrosis, and calcification [31]. These cell populations may remodel tissue matrices, regulate fibrosis, and structural repair. Furthermore, active recruitment of circulating monocytes into the valve is increased with age, promoting differentiation into macrophages and degenerative valvular changes. In turn, although cross-linking of IgE-FcεRI complexes on monocytes might increase secretion of pro inflammatory tumor necrosis factor α (TNFα) and interleukin-6 (IL-6), it also significantly upregulates the production of IL-10, limiting the further development of inflammation. Moreover, IgE binding could critically impair these cells’ phagocytic function, a primary mechanism contributing to the tissue injury repair and extracellular matrix remodeling [32,33]. It is worth mentioning that patients with more severe AS are characterized by increased circulating monocyte numbers, which interestingly correlate with AVA [34]. Thus, monocyte-related inflammation seems to be critical in a more severe stage of the disease. Our outcomes indicate an auxiliary role of IgE network in this regulation, although extensive observational and experimental studies are needed to verify the above hypothesis. The findings and the speculations potentially providing them with some mechanistic explanations are presented in Figure 3.

Another possible explanation of the observed relationship might be a linkage disequilibrium between rs2251746 polymorphism and genetic variants in another gene localized distantly from *FCER1A* and not directly related to the IgE network. For example, a strong correlation was observed between a *FCER1A* polymorphism belonging to the same tagging bin as rs2251746 and the expression of *OR10J5*, a gene located about 250 kb upstream [35]. One cannot exclude a similar relationship between rs2251746 and another gene on chromosome 1, which is not directly related to the IgE network but has anti-inflammatory or immune modulating properties, slowing the AS progression. In this scenario, the effect of the rs2251746 polymorphism on serum IgE would be functionally meaningless, at least in the context of the AS severity, although higher total serum IgE could be used as a biomarker of the less severe AS. The contribution of other mechanisms, however, cannot be excluded.

Our study has several limitations. Most importantly, total serum IgE was not measured in all analyzed subjects. The reason behind this was the design of the study, with the original group (Table S1) being a screening group for detection of the association between IgE network and acquired AS severity and the expanded group (Table S2) designated for genetic association testing without IgE level analysis. Other limitations, such as a lack of a replication group or functional studies, make our results preliminary, matching the type of this report, i.e., communication.

## 5. Conclusions

We found for the first time the relationships between the elements of the IgE network and acquired AS severity. Specifically, higher AVA corresponding to the less advanced AS was associated with elevated total serum IgE levels or the major allele of *FCER1A* rs2251746 polymorphism. This intriguing observation is, however, preliminary and requires further investigation. Moreover, the mechanisms underlying our current results need to be elucidated.

## Figures and Tables

**Table 1:** Correlations of total serum immunoglobulin E (IgE) levels (IU/mL) with echocardiographic parameters in patients with acquired aortic stenosis (*n* = 115).

Echocardiographic Parameter	Number of Data Pairs	Spearman’s Rank Correlation Coefficient (*R*)	*P*-Value
Mean aortic gradient, mm Hg	100	−0.11	0.28
Maximum aortic gradient, mm Hg	95	−0.06	0.55
Left ventricular ejection fraction, %	101	−0.03	0.75
Aortic valve area, cm^2^	82	0.27	0.01

## Data Availability

Data available on request.

## Supplementary Materials

The following are available online at https://www.mdpi.com/2227-9059/9/1/23/s1: Table S1: The original group of aortic stenosis (AS) patients. Table S2: The expanded group of aortic stenosis (AS) patients. Table S3: Genotyping of the gene encoding the α-subunit of the high-affinity IgE receptor (*FCER1A*) polymorphisms. Table S4: The α-subunit of the high-affinity IgE receptor gene (*FCER1A*) polymorphisms and total serum immunoglobulin E (IgE) levels in aortic stenosis (AS) patients.

## References

[1] Lippi G., Cervellin G., Sanchis-Gomar F. (2014). Immunoglobulin E (IgE) and ischemic heart disease. Which came first, the chicken or the egg?. Ann. Med..

[2] Potaczek D.P. (2014). Links between allergy and cardiovascular or hemostatic system. Int. J. Cardiol..

[3] Hermans M., Roeters van Lennep J., van Daele P., Bot I. (2019). Mast Cells in Cardiovascular Disease: From Bench to Bedside. Int. J. Mol. Sci..

[4] Potaczek D.P., Kabesch M. (2012). Current concepts of IgE regulation and impact of genetic determinants. Clin. Exp. Allergy.

[5] Gould H.J., Sutton B.J. (2008). IgE in allergy and asthma today. Nat. Rev. Immunol..

[6] Guo X., Yuan S., Liu Y., Zeng Y., Xie H., Liu Z., Zhang S., Fang Q., Wang J., Shen Z. (2016). Serum IgE levels are associated with coronary artery disease severity. Atherosclerosis.

[7] Kounis N.G., Hahalis G. (2016). Serum IgE levels in coronary artery disease. Atherosclerosis.

[8] Wang J., Cheng X., Xiang M.X., Alanne-Kinnunen M., Wang J.A., Chen H., He A., Sun X., Lin Y., Tang T.T. (2011). IgE stimulates human and mouse arterial cell apoptosis and cytokine expression and promotes atherogenesis in Apoe^−/−^ mice. J. Clin. Investig..

[9] Kostyunin A., Mukhamadiyarov R., Glushkova T., Bogdanov L., Shishkova D., Osyaev N., Ovcharenko E., Kutikhin A. (2020). Ultrastructural Pathology of Atherosclerosis, Calcific Aortic Valve Disease, and Bioprosthetic Heart Valve Degeneration: Commonalities and Differences. Int. J. Mol. Sci..

[10] De Oliveira Sá M.P.B., Cavalcanti L.R.P., Perazzo Á.M., Gomes R.A.F., Clavel M.A., Pibarot P., Biondi-Zoccai G., Zhigalov K., Weymann A., Ruhparwar A. (2020). Calcific Aortic Valve Stenosis and Atherosclerotic Calcification. Curr. Atheroscler. Rep..

[11] Min K.B., Min J.Y. (2019). Risk of Cardiovascular Mortality in Relation to Increased Total Serum IgE Levels in Older Adults: A Population-Based Cohort Study. Int. J. Environ. Res. Public Health.

[12] Donato M., Ferri N., Lupo M.G., Faggin E., Rattazzi M. (2020). Current Evidence and Future Perspectives on Pharmacological Treatment of Calcific Aortic Valve Stenosis. Int. J. Mol. Sci..

[13] Granada M., Wilk J.B., Tuzova M., Strachan D.P., Weidinger S., Albrecht E., Gieger C., Heinrich J., Himes B.E., Hunninghake G.M. (2012). A genome-wide association study of plasma total IgE concentrations in the Framingham Heart Study. J. Allergy Clin. Immunol..

[14] Sharma V., Michel S., Gaertner V., Franke A., Vogelberg C., von Berg A., Bufe A., Heinzmann A., Laub O., Rietschel E. (2014). Fine-mapping of IgE-associated loci 1q23, 5q31, and 12q13 using 1000 Genomes Project data. Allergy.

[15] Weidinger S., Gieger C., Rodriguez E., Baurecht H., Mempel M., Klopp N., Gohlke H., Wagenpfeil S., Ollert M., Ring J. (2008). Genome-wide scan on total serum IgE levels identifies FCER1A as novel susceptibility locus. PLoS Genet..

[16] Potaczek D.P., Michel S., Sharma V., Zeilinger S., Vogelberg C., von Berg A., Bufe A., Heinzmann A., Laub O., Rietschel E. (2013). Different FCER1A polymorphisms influence IgE levels in asthmatics and non-asthmatics. Pediatr. Allergy Immunol..

[17] Wypasek E., Potaczek D.P., Lamplmayr M., Sadowski J., Undas A. (2014). Interleukin-6 receptor Asp358Ala gene polymorphism is associated with plasma C-reactive protein levels and severity of aortic valve stenosis. Clin. Chem. Lab. Med..

[18] Wypasek E., Potaczek D.P., Undas A. (2015). Association of the C-Reactive Protein Gene (CRP) rs1205 C>T Polymorphism with Aortic Valve Calcification in Patients with Aortic Stenosis. Int. J. Mol. Sci..

[19] Potaczek D.P., Przytulska-Szczerbik A., Bazan-Socha S., Nastałek M., Wojas-Pelc A., Okumura K., Nishiyama C., Jurczyszyn A., Undas A., Wypasek E. (2020). Interaction between functional polymorphisms in FCER1A and TLR2 and the severity of atopic dermatitis. Hum. Immunol..

[20] Teirstein P., Yeager M., Yock P.G., Popp R.L. (1986). Doppler echocardiographic measurement of aortic valve area in aortic stenosis: A noninvasive application of the Gorlin formula. J. Am. Coll. Cardiol..

[21] Hellman L. (2007). Regulation of IgE homeostasis, and the identification of potential targets for therapeutic intervention. Biomed. Pharm..

[22] Potaczek D.P., Nishiyama C., Sanak M., Szczeklik A., Okumura K. (2009). Genetic variability of the high-affinity IgE receptor α-subunit (FcepsilonRIalpha). Immunol. Res..

[23] Hasegawa M., Nishiyama C., Nishiyama M., Akizawa Y., Mitsuishi K., Ito T., Kawada H., Furukawa S., Ra C., Okumura K. (2003). A novel -66T/C polymorphism in Fc epsilon RI α-chain promoter affecting the transcription activity: Possible relationship to allergic diseases. J. Immunol..

[24] Kanada S., Nakano N., Potaczek D.P., Maeda K., Shimokawa N., Niwa Y., Fukai T., Sanak M., Szczeklik A., Yagita H. (2008). Two different transcription factors discriminate the -315C>T polymorphism of the Fc epsilon *RIα* gene: Binding of Sp1 to -315C and of a high mobility group-related molecule to -315T. J. Immunol..

[25] Wypasek E., Natorska J., Grudzień G., Filip G., Sadowski J., Undas A. (2013). Mast cells in human stenotic aortic valves are associated with the severity of stenosis. Inflammation.

[26] Kounis N.G., Mazarakis A., Tsigkas G., Giannopoulos S., Goudevenos J. (2011). Kounis syndrome: A new twist on an old disease. Future Cardiol..

[27] Kounis N.G., Koniari I., Velissaris D., Tzanis G., Hahalis G. (2019). Kounis Syndrome—Not a Single-organ Arterial Disorder but a Multisystem and Multidisciplinary Disease. Balkan Med. J..

[28] Varricchi G., de Paulis A., Marone G., Galli S.J. (2019). Future Needs in Mast Cell Biology. Int. J. Mol. Sci..

[29] Babina M., Guhl S., Artuc M., Zuberbier T. (2018). Allergic FcεRI- and pseudo-allergic MRGPRX2-triggered mast cell activation routes are independent and inversely regulated by SCF. Allergy.

[30] Wang Z., Guhl S., Franke K., Artuc M., Zuberbier T., Babina M. (2019). IL-33 and MRGPRX2-Triggered Activation of Human Skin Mast Cells-Elimination of Receptor Expression on Chronic Exposure, but Reinforced Degranulation on Acute Priming. Cells.

[31] Sraeyes S., Pham D.H., Gee T.W., Hua J., Butcher J.T. (2018). Monocytes and Macrophages in Heart Valves: Uninvited Guests or Critical Performers?. Curr. Opin. Biomed. Eng..

[32] Pyle D.M., Yang V.S., Gruchalla R.S., Farrar J.D., Gill M.A. (2013). IgE cross-linking critically impairs human monocyte function by blocking phagocytosis. J. Allergy Clin. Immunol..

[33] Rowe R.K., Pyle D.M., Tomlinson A.R., Lv T., Hu Z., Gill M.A. (2017). IgE cross-linking impairs monocyte antiviral responses and inhibits influenza-driven TH1 differentiation. J. Allergy Clin. Immunol..

[34] Shimoni S., Meledin V., Bar I., Fabricant J., Gandelman G., George J. (2016). Circulating CD14^+^ monocytes in patients with aortic stenosis. J. Geriatr. Cardiol..

[35] Ferreira M.A.R., Vonk J.M., Baurecht H., Marenholz I., Tian C., Hoffman J.D., Helmer Q., Tillander A., Ullemar V., Lu Y. (2019). Eleven loci with new reproducible genetic associations with allergic disease risk. J. Allergy Clin. Immunol..

