# Supplementary material for: Elements of Immunoglobulin E Network Associate with Aortic Valve Area in Patients with Acquired Aortic Stenosis
Source: Biomedicines. 2020 Dec 31;9(1):23. doi: 10.3390/biomedicines9010023 (PMC7824289; doi:10.3390/biomedicines9010023)
Supplement: Supplementary file 1 [file biomedicines-09-00023-s001.pdf]

Table S1. The original group of aortic stenosis (AS) patients.

	**AS Patients (*n* = 115)**
Age, years	65.7 ± 9.7
Male gender, *n* (%)	73 (63.5)
BMI, kg/m^2	29.1 ± 5.4<br>*n* = 88
Mean gradient, mmHg	50.0 [43.2-60.5]<br>*n* = 100
Maximum gradient, mmHg	86.0 [73.3-100.8]<br>*n* = 95
LVEF, %	60.0 [51.5-65.0]<br>*n* = 101
AVA, cm^2	0.78 [0.65-0.90]<br>*n* = 82
Plasma CRP, mg/L	1.32 [0.89-2.93]<br>*n* = 113
Total serum IgE, IU/mL	26.5 [0.5-66.2]

BMI, body mass index; LVEF, left ventricular ejection fraction; AVA, aortic valve area; CRP, C-reactive protein; IgE, immunoglobulin E. Quantitative data are presented as mean ± standard deviation or median [interquartile range].

Table S2. The expanded group of aortic stenosis (AS) patients.*

	**AS Patients (*n* = 420)***
Age, years*	64.8 ± 10.7
Male gender, *n* (%)*	235 (56.0)
BMI, kg/m^2	27.8 ± 4.4<br>*n* = 393
Mean gradient, mmHg*	50.0 [39.0-63.7]<br>*n* = 401
Maximum gradient, mmHg*	84.7 [65.1-102.0]<br>*n* = 399
LVEF, %*	62.7 [55.0-68.0]<br>*n* = 406
AVA, cm^2*	0.74 [0.60-0.93]<br>*n* = 385
Plasma CRP, mg/L*	1.94 [1.09-3.61]<br>*n* = 414

For abbreviations and legends, please, refer to Table S1. *All subjects representing the original group of 115 AS subjects in whom total serum IgE was analyzed (see Table S1) and the additional 305 AS subjects participating in the previous genetic studies [1,2] comprised the expanded group of AS patients.

Table S3. Genotyping of the gene encoding the α-subunit of the high-affinity IgE receptor (*FCER1A*) polymorphisms.

**Polymorphism**	**Individuals Tested, *n****	**Call Rate, %**	**Genotypes**			**MAF, %**	**HWE, *p*-Value‡**
			**Major Homozygote, n (%)†**	**Heterozygote, n (%)†**	**Minor Homozygote, n (%)†**		
rs2251746	406	98.0	TT, 213 (53.5)	TC, 157 (39.4)	CC, 28 (7.0)	26.8	0.99
rs2252226	406	98.0	TT, 131 (32.9)	TC, 197 (49.5)	CC, 70 (17.6)	42.3	0.96

MAF, minor allele frequency; HWE, Hardy–Weinberg equilibrium. * Sufficient genomic DNA of satisfying quality was available in 406 out of 420 subjects comprising the expanded group of aortic stenosis patients (see Table S2). † The percentages refer to the number of successfully genotyped individuals for whom sufficient genomic DNA of satisfying quality was available. ‡ HWE *p*-values were calculated using chi-squared goodness-of-fit test.

Table S4. The α-subunit of the high-affinity IgE receptor gene (*FCER1A*) polymorphisms and total serum immunoglobulin E (IgE) levels in aortic stenosis patients.

					***p*-Value**			
rs2251746								
	Genotype (*n*)	TT (*n* = 66)	TC (*n* = 41)	CC (*n* = 8)	TT vs. TC vs. CC*	TT + TC vs. CC†	TT vs. TC + CC†	TT vs. CC†
	Total serum IgE, IU/mL	32.5 [0.5-73.3]	26.8 [0.5-67.1]	6.4 [0.5-17.2]	0.09	0.03	0.25	0.03
rs2252226								
	Genotype (*n*)	TT (*n* = 27)	TC (*n* = 60)	CC (*n* = 28)	TT vs. TC vs. CC*	TT + TC vs. CC†	TT vs. TC + CC†	TT vs. CC†
	Total serum IgE, IU/mL	12.2 [0.5-39.5]	28.1 [3.3-69.9]	34.0 [0.5-87.7]	0.19	0.47	0.07	0.14

IgE values are given as median [interquartile range]. * *p*-values calculated using Kruskal–Wallis test. † *p*-values calculated using Mann–Whitney test.

References

1. Wypasek E, Potaczek DP, Lamplmayr M, Sadowski J, Undas A. Interleukin-6 receptor Asp358Ala gene polymorphism is associated with plasma C-reactive protein levels and severity of aortic valve stenosis. *Clin Chem Lab Med* 2014;52:1049-1056. doi: 10.1515/cclm-2013-0606.
2. Wypasek E, Potaczek DP, Undas A. Association of the C-Reactive Protein Gene (CRP) rs1205 C>T Polymorphism with Aortic Valve Calcification in Patients with Aortic Stenosis. *Int J Mol Sci* 2015;16:23745-23759. doi: 10.3390/ijms161023745.
